# Sub-microscopic infections and long-term recrudescence of *Plasmodium falciparum *in Mozambican pregnant women

**DOI:** 10.1186/1475-2875-8-9

**Published:** 2009-01-09

**Authors:** Alfredo Mayor, Elisa Serra-Casas, Azucena Bardají, Sergi Sanz, Laura Puyol, Pau Cisteró, Betuel Sigauque, Inacio Mandomando, John J Aponte, Pedro L Alonso, Clara Menéndez

**Affiliations:** 1 Centre de Recerca en Salut Internacional de Barcelona, Hospital Clínic/Institut d'Investigacions Biomèdiques August Pi i Sunyer, Universitat de Barcelona, Rosselló 132, E-08036 Barcelona, Spain; 2 Centro de Investigação em Saúde da Manhiça (CISM), Maputo, Mozambique; 3 Direcção Nacional de Saúde/Instituto Nacional de Saúde, Ministerio de Saúde, Maputo, Mozambique

## Abstract

**Background:**

Control of malaria in pregnancy remains a public health challenge. Improvements in its correct diagnosis and the adequacy of protocols to evaluate anti-malarial drug efficacy in pregnancy, are essential to achieve this goal.

**Methods:**

The presence of *Plasmodium falciparum *was assessed by real-time (RT) PCR in 284 blood samples from pregnant women with clinical complaints suggestive of malaria, attending the maternity clinic of a Mozambican rural hospital. Parasite recrudescences in 33 consecutive paired episodes during the same pregnancy were identified by *msp1 *and *msp2 *genotyping.

**Results:**

Prevalence of parasitaemia by microscopy was 5.3% (15/284) and 23.2% (66/284) by RT-PCR. Sensitivity of microscopy, compared to RT-PCR detection, was 22.7%. Risk of maternal anaemia was higher in PCR-positive women than in PCR-negative women (odds ratio [OR] = 1.92, 95% confidence interval [CI] 1.09–3.36). Genotyping confirmed that recrudescence after malaria treatment occurred in 7 (21%) out of 33 pregnant women with consecutive episodes during the same pregnancy (time range between recrudescent episodes: 14 to 187 days).

**Conclusion:**

More accurate and sensitive diagnostic indicators of malaria infection in pregnancy are needed to improve malaria control. Longer follow-up periods than the standard in vivo drug efficacy protocol should be used to assess anti-malarial drug efficacy in pregnancy.

## Background

Pregnant women are at higher risk of *Plasmodium falciparum *infection and disease [1], frequently manifested as maternal anaemia, pre-term delivery and low birth weight [2,3]. Infections during pregnancy need to be eliminated with effective anti-malarials to reduce the burden of disease in mothers and their children. Current guidelines to control malaria in pregnancy in sub-Saharan Africa consist of prompt and effective case management of malaria illness, combined with prevention of infection and/or disease through insecticide-treated nets (ITNs) and intermittent preventive treatment (IPTp) [4].

Control of malaria in pregnancy remains a challenge due to a number of factors. The low specificity of signs and symptoms [5,6] and the limitations of the techniques to detect *P. falciparum *infections impose serious difficulties in the identification of malaria episodes. Until recently, malaria diagnosis relied on the microscopic detection of *Plasmodium *on Giemsa-stained blood smears. Lately, circulating antigen [7] and polymerase chain reaction (PCR)-based diagnostic methods [8-12] have been added as malaria diagnostic tools. In malaria endemic areas, PCR allows the identification of a high proportion of pregnant women with *P. falciparum *parasitaemia levels below the threshold of microscopy [8-12]. Real-time PCR (RT-PCR), a more sensitive and specific detection methodology [13,14], has been shown to be useful in predicting adverse outcomes of malaria in pregnancy [15,16].

The declining efficacy of classically recommended anti-malarial drugs due to the increase of anti-malarial-resistant *P. falciparum *parasites [17], results in multiple treatment failures and prolonged parasitization of the foeto-placental unit with the subsequent adverse events on mother and child health. New anti-malarial drug combinations are now being recommended to replace the less effective ones for the general population. However, there is limited or non-existent information on the safety and efficacy of these new drug alternatives in pregnancy. Data obtained from in vivo studies in children may not be appropriated for determining anti-malarial treatment efficacy in pregnant women because of differences in host immunity, as well as pharmacokinetics [18-21]. Critical analysis of the in vivo standard methods to measure drug efficacy is essential to correctly monitor drug resistance and evaluate new anti-malarials to be administered in pregnancy.

In this study, a PCR-based approach to assess the impact of sub-microscopic infections on disease manifestation of pregnant women attending the maternity clinic of a rural hospital in Mozambique was used. Genetic characterization of parasites isolated from consecutive episodes during the same pregnancy allowed to determine the extent of parasite recrudescences after anti-malarial treatment.

## Methods

### Study area and population

The study was carried out at the Centro de Investigação em Saúde da Manhiça (CISM) in Manhiça District, southern Mozambique. Adjacent to the CISM is the Manhiça District Hospital (MDH), a 110 bed health facility, which provides curative and preventive services. The characteristics of the area have been described in detail elsewhere [22]. Perennial malaria transmission with some seasonality is mostly attributed to *P. falciparum*. *Anopheles funestus *is the main vector, and the estimated entomological inoculation rate for 2002 was 38 infective bites per person per year [23]. The most recent data on the efficacy of sulphadoxine-pyrimethamine (SP) and chloroquine (CQ) in children in this area showed a combined (early and late) treatment failure rate of 53% for CQ and 17.3% for SP [24].

### Study design

This study was conducted in the context of a health facility-based descriptive study that aimed to characterize the clinical presentation of malaria in Mozambican pregnant women and to evaluate the adequacy of case management based on clinical complaints [6]. A round-the-clock morbidity surveillance system was established at the Maternity Clinic of the Manhica District Hospital (MDH), as a passive case detection system, for all women attending this clinic with clinical complaints. Pregnant women attending the Maternity Clinic between August 2003 and November 2005, with clinical complaints and who gave verbal informed consent, were asked for information on age, parity, gestational age, and signs and symptoms focused on malaria (axillary temperature ≥ 37.5°C, referred history of fever in the last 24 hours, pallor, arthromyalgias, headache and/or history of convulsions). This clinical and demographic information was collected onto a standardized questionnaire. A capillary blood sample for quantification of *P. falciparum *parasitaemia and haematocrit, and blood on DNA filter paper (Schleicher & Schuell number 903™; Dassel, Germany) were collected if at least one of the pre-defined clinical criteria suggestive of malaria was met. A random selection of 10% of the filter papers collected, and all the available filter papers from women with two consecutive malaria episodes during the same pregnancy, were analysed by RT-PCR.

Following national guidelines at the time of the study, women were treated with SP plus CQ in case of non-complicated malaria, except for the first trimester when CQ alone was given. Oral quinine (Q) for seven days was given as rescue treatment. Women with complicated malaria were admitted at the maternity ward, and treated with parenteral quinine followed by SP. Anaemia was treated with oral ferrous sulphate and folic acid for one month. The study protocol was approved by the National Mozambican Ethics Committee and the Hospital Clinic of Barcelona Ethics Review Committee.

### Laboratory methods

Thick and thin blood films, in duplicate, were stained with Giemsa and read to determine parasite species and density of *P. falciparum* asexual stages per 200 leukocytes, according to standard, quality-controlled procedures [25]. The haematocrit was measured using a microhaematocrit centrifuge and read in a Hawksley (Lancing, UK) haematocrit reader.

### Detection of *Plasmodium falciparum *by real time PCR analysis

Parasite DNA was extracted from 50 μl blood on filter paper by using the ABIPrism 6700 Automated Nucleic Acid Work Station (Applied Biosystems), according to the manufacturer's instructions. Negative extraction controls consisting of water or non-infected erythrocytes were included in the process. Purified DNA templates were amplified in an ABI PRISM 7900 HT Real-Time System (Applied Biosystems) following a previously described method [13]. Briefly, a 20 μl PCR mixture was performed using 5 μl of template, 10 μl of 2 × TaqMan^® ^Universal PCR Master Mix (Applied Biosystems), a 300 nM concentration of each primers specific for 18S ribosomal RNA gene of *P. falciparum*, and a 150 nM concentration of probe labelled with 6-carboxy-fluorescein (FAM) as a reporter and 6-carboxytetramethylrhodamine (TAMRA) as a quencher. Amplification and detection were performed under the following conditions: 2 min at 50°C, 10 min at 95°C, and 40 cycles of 15 s at 95°C and 1 min at 60°C. The results were automatically analysed by the ABI Prism SDS2.1 software. Each specimen was run in duplicate. A standard curve was prepared from titrated samples containing known numbers of ring-infected erythrocytes diluted in whole blood. The highest dilution of the curve that gave reproducible results in all the optimization assays was the equivalent to 50 parasites per ml of blood. The standard curve was run in triplicate for each test. RT-PCR efficiency was validated by comparing the slope of the standard curve to the theoretical optimum of -3.32 which reflects 100% efficient amplification, and was found to be -3.21 ± 0.13, R^2 ^= 0.99 (*n *= 5).

### Genotyping of *P. falciparum *isolates

*Plasmodium falciparum *infections from consecutive malaria episodes during the same pregnancy were characterized on the basis of the fragment size of the amplified *msp1 *(allelic families MAD20, K1 and RO33) and *msp2 *(allelic families FC27 and 3D7/IC1) loci using a previously described PCR approach [26]. Each band was considered an individual strain. Strains were considered the same if the band had an identical size as determined by migration in a 2.5% agarose gel. Products from episodes of the same women were run side-by-side to allow for direct size comparison. The frequency distribution of the *msp1 *and *mp2 *alleles circulating in the study area was calculated by measuring the proportion of each detected genotype, as defined into 20-base pair ranges, in 60 isolates from pregnant women [27].

### Definitions and statistical methods

A malaria episode was defined as a *P. falciparum *parasitaemia of any density, and presence of any sign and/or symptom suggestive of malaria (presence or history of fever in the last 24 hours, headache, arthromyalgias, convulsions, pallor) [6]. Fever was defined as a temperature equal or higher to 37.5°C, and anaemia as a haematocrit less than 33%. Peripheral blood *P. falciparum *infections were grouped into the following categories: a) microscopically confirmed parasitaemia; b) RT-PCR confirmed parasitaemia; c) sub-microscopic infections (negative samples by microscopic examination which gave a RT-PCR positive result); d) no infection (negative by microscopy and RT-PCR). In this study, the duration of a single malaria episode was estimated as 28 days so as to differentiate between visits on the same episode and a new episode. Multiplicity of infection (MOI) was defined as the maximum number of *msp1/msp2*-amplicons detected in the sample. To eliminate the possibility of misclassifying patients with common *msp1 *and *msp2 *variants as treatment failures, recrudescence was only considered when all strains in the second episode were present in the previous episode, and no new genotypes were found (i.e., both *msp1 *and *msp2 *amplification products from the second episode were observed in the previous episode). Infections in the second episode that consisted on different strains not present in the previous episode were designated as new infections. When the second malaria episode contained strains present in the previous episode but also new strains, infections were categorized as mixed [28].

Double data entry, validation, and cleaning were done using Microsoft Visual FoxPro 5.0, and statistical analysis was performed using STATA.9 (STATA corporation, College Station, TX, USA). To evaluate the association between the presence of signs and symptoms suggestive of malaria, age, parity, gestational age, number of previous malaria episodes and season with parasitaemia, univariate logistic regression models were used. Sensitivity and specificity of microscopic determination was calculated considering RT-PCR as the gold standard. The cumulative probability of two parasites in two independent episodes being by chance the same for a particular 2-loci genotype was calculated from the frequencies of *msp1 *and *msp2 *genotypes detected in 60 pregnant women [27]. Days between episodes and multiplicity of infection (MOI) were compared by t Student's test. Monte Carlo permutation test with 1,000 random permutations was performed for each comparison to obtain a more robust estimation of the *p*-value [29]. A permutated *p*-value < 0.05 was considered statistically significant.

## Results

### Detection of malaria infection

During the study period (August 2003–November 2005), 3,129 filter papers corresponding to first visits with criteria for blood collection and complete laboratory results were collected from pregnant women attending the MDH. Three hundred out of these samples were randomly selected. Sixteen filter papers were not found. Among the women from whom the 284 filter papers were available, 70.9% (200/282) were 20–34 years of age, 59.4% (168/283) were in their second to fourth pregnancies, and 48.2% (137/284) had anaemia.

Of the 284 women, 15 (5.3%) and 66 (23.2%) had malaria parasitaemia as detected by microscopy and RT-PCR, respectively. Fifty-one (18.9%) of the 269 women found to be aparasitaemic by microscopy were positive by RT-PCR (sub-microscopic infections). Compared with RT-PCR detection, microscopy showed a sensitivity of 22.7%. Prevalence of *P. falciparum *malaria, both by microscopy and RT-PCR, was equal across age and parity groups (Table 1 and Table 2). Prevalence of sub-microscopic infections (n = 51) was not statistically different across maternal age (p = 1.00), gestational age (p = 1.00) and gravidity group (p = 0.68). Infections detected by RT-PCR were more prevalent among women in the third trimester of pregnancy than among those in the first and second trimester (Table 2), although estimates were not statistically significant (p = 0.082). This trend was not found in infections detected by microscopy (Table 1).

**Table 1:** Association between parasitaemia, as detected by microscopy, and mother age, pregnancy status and signs/symptoms of malaria.

		**Microscopy**						
					
		**negative** n = 269		**positive** n = 15				
					
**Variable**		**n**	**%**	**n**	**%**	**OR**	**95% CI**	p-value*
Age**	<20 y	50	19	4	27	1		0.606
	20–34 y	190	71	10	67	0.66	(0.20; 2.19)	
	>= 35 y	27	10	1	7	0.46	(0.05; 4.35)	
Gestational age (weeks)	1–12	74	28	3	20	1		0.670
	13 – 24	68	25	3	20	1.09	(0.21; 5.58)	
	> 24	127	47	9	60	1.75	(0.46; 6.66)	
Parity***	0	32	12	0	0	-		0.292
	1–3	156	58	12	80	1		
	>= 4	80	30	3	20	0.49	(0.13; 1.78)	
Season	Dry	132	49	11	73	1		0.105
	Wet	137	51	4	27	0.35	(0.11; 1.13)	
Fever	No	248	92	13	87	1		0.481
	Yes	21	8	2	13	1.82	(0.38; 8.59)	
Fever/history of fever***	No	107	40	4	27	1		0.264
	Yes	161	60	11	73	1.83	(0.57; 5.89)	
Arthromyalgia	No	63	23	3	20	1		0.767
	Yes	206	77	12	80	1.22	(0.33; 4.47)	
Headache	No	27	10	4	27	1		0.089
	Yes	242	90	11	73	0.31	(0.09; 1.03)	
Pallor	No	255	95	14	93	1		1.000
	Yes	14	5	1	7	1.3	(0.16; 10.61)	
Anemia	No	137	51	10	67	1		0.288
	Yes	132	49	5	33	0.52	(0.17; 1.56)	

*, Univariate logistic regression model; **, Two data missing; ***, One data missing; OR, Odds ratio; CI, Confidence interval.

**Table 2:** Association between parasitaemia, as detected by RT-PCR, and mother age, pregnancy status and signs/symptoms of malaria.

		**PCR**						
					
		**negative** n = 218		**positive** n = 66				
					
**Variable**		**n**	**%**	**n**	**%**	**OR**	**95% CI**	p-value*
Age**	<20 y	38	18	16	24	1		0.480
	20–34 y	156	72	44	67	0.67	(0.34; 1.31)	
	>= 35 y	22	10	6	9	0.65	(0.22; 1.90)	
Gestational age (weeks)	1–12	64	29	13	20	1		0.082
	13 – 24	58	27	13	20	1.10	(0.47; 2.57)	
	> 24	96	44	40	61	2.05	(1.02; 4.14)	
Parity***	0	28	13	4	6	1		0.094
	1–3	122	56	46	70	2.64	(0.88; 7.94)	
	>= 4	67	31	16	24	1.67	(0.51; 5.45)	
Season	Dry	108	50	35	53	1		0.684
	Wet	110	50	31	47	0.87	(0.50; 1.51)	
Fever	No	200	92	61	92	1		1.000
	Yes	18	8	5	8	0.91	(0.32; 2.55)	
Fever/history of fever***	No	84	39	27	41	1		0.772
	Yes	133	61	39	59	0.91	(0.52; 1.60)	
Arthromyalgia	No	55	25	11	17	1		0.176
	Yes	163	75	55	83	1.69	(0.82; 3.45)	
Headache	No	24	11	7	11	1		1.000
	Yes	194	89	59	89	1.04	(0.43; 2.54)	
Pallor	No	205	94	64	97	1		0.568
	Yes	13	6	2	3	0.49	(0.11; 2.24)	
Anemia	No	121	56	26	39	1		0.026
	Yes	97	44	40	61	1.92	(1.09; 3.36)	

*, Univariate logistic regression model; **, Two data missing; ***, One data missing; OR, Odds ratio; CI, Confidence interval.

### Malaria infection and clinical presentation

There was no evidence of an association between signs or symptoms suggestive of malaria and the presence of microscopically detected peripheral malaria (Table 1). Risk of maternal anaemia was higher in women with RT-PCR detected parasitaemina than in women with a negative RT-PCR result (Table 2, OR = 1.92, 95% CI 1.09–3.36, p = 0.026). Women with sub-microscopic infections also had a statistically significant higher risk of anaemia compared with women with negative microscopy and RT-PCR test (OR = 2.80, 95% CI 1.46–5.38, p = 0.002). No other malaria sign or symptom was found to be associated with PCR-detected or sub-microscopic infections.

### Malaria recrudescence in pregnancy

**Figure 1:**
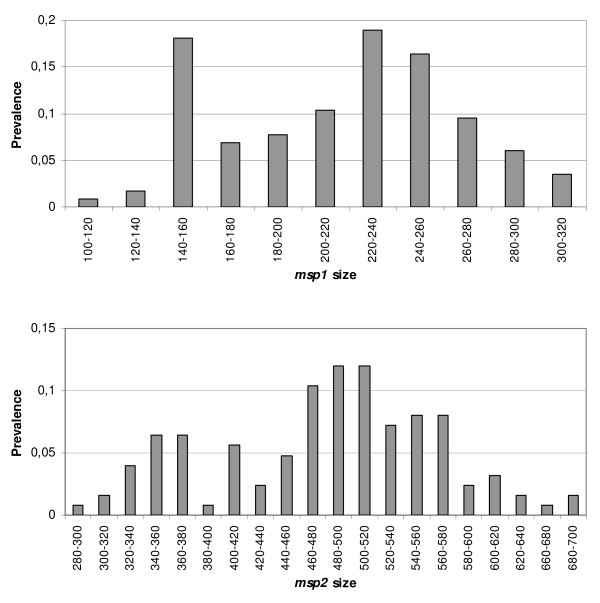
**Prevalence of *msp1 *and *msp2 *size variants (in base pairs) in *P. falciparum *parasite population from Manhiça**.

Eleven *msp1 *and 20 *msp2 *genotypes were found in 60 pregnant women, with allelic frequencies ranging from 0.008 to 0.18 (Figure 1). Four *msp1 *and 3 *msp2 *variants were present at frequencies higher than 10%. The cumulative probability of two independent strains being by chance the same for *msp1 *and *msp2 *in two different episodes was 0.99%. Given this low probability of repeated occurrence by chance of a particular two-loci genotype in the same patient, coincidence of both *msp1 *and *msp2 *in consecutive episodes was considered evidence of the same parasite.

Sixty-six filter papers were analysed from 33 women with two consecutive malaria episodes during the same pregnancy (Table 3). Recrudescent infections (isolates sharing the same *msp1 *and *msp2 *genotypes with no evidence of new infections) occurred in 7 (21.2%) of the 33 women. In 22 (66.7%) of the women, the second episode consisted of *P. falciparum *isolates with *msp1 *and *msp2 *genotypes not present in the previous episodes (new infections). Mixed infections (both recrudescent and new strains present) were present in 4 (12.1%) of the women. Mean duration of the interval between consecutive episodes was similar in recrudescent (58 days, SD 57.7, range 14–187) and new infections (61.2 days, SD 30, range 20–129) (p = 0.84). No statistical difference was found in the mean multiplicity of infection (MOI) from first (1.95, SD 0.89) and second episodes (1.58, SD 0.75) (p = 0.33), and from recrudescent (1.71, SD 0.48) and new infections (1.76, SD 0.81) (p = 0.82).

**Table 3:** Malaria genotypes and multiplicity of infection detected in 33 consecutive paired episodes during the same pregnancy.

			***msp1*/*msp2 *genotypes**			
					
	**MOI**		**Same in**	**Different in**		
					
**Infection**	**Episode 1**	**Episode 2**	**both episodes**	**episode 2**	**Tx**	**Days**
**New**						
	2	1	0	1	CQ	20
	1	1	0	1	CQ	25
	2	1	0	1	CQ+SP	31
	1	3	0	3	CQ	32
	2	2	0	2	CQ+SP	35
	3	1	0	1	CQ+SP	41
	3	4	0	4	CQ	47
	1	2	0	2	CQ+SP	47
	2	1	0	1	Q+SP	49
	2	1	0	1	CQ	50
	2	2	0	2	Q+SP	52
	4	2	0	2	CQ	55
	1	1	0	1	CQ+SP	56
	1	1	0	1	CQ	59
	2	1	0	1	CQ+SP	64
	1	2	0	2	CQ+SP	72
	2	1	0	1	CQ+SP	77
	1	2	0	2	CQ+SP	81
	4	2	0	2	CQ+SP	93
	2	2	0	2	CQ+SP	116
	2	2	0	2	CQ+SP	117
	2	1	0	1	CQ	129
**Recrudescent**						
	1	1	1	0	CQ	14
	3	2	2	0	CQ	39
	2	2	2	0	CQ+SP	41
	3	2	2	0	CQ+SP	41
	1	1	1	0	CQ+SP	42
	2	2	2	0	CQ	42
	3	2	2	0	Q	187
**Mixed**						
	1	2	1	1	Q	29
	3	2	1	1	CQ+SP	38
	3	3	1	2	CQ	98
	3	3	1	2	CQ	122

MOI, Multiplicity of infection; Tx, anti-malarial used to treat episode 1; Days, Days between epidodes; Q, Quinine; CQ, Chloroquine; SP, Sulphadoxine-Pyrimethamine.

## Discussion

This study described the prevalence of *P. falciparum *infection detected by real time PCR among Mozambican pregnant women attending a rural maternity clinic with clinical complaints suggestive of malaria. These results showed that microscopically detectable *P. falciparum *parasitaemia in peripheral blood is a rather poor indicator of the actual presence of infection in pregnancy, as suggested by the low sensitivity of microscopy as compared to RT-PCR (22.7%) and the high prevalence of sub-microscopically infected women (18.9%). In other settings, sub-patent malaria infections have been found in up to 55% of the pregnant women [8-12]. This suggests the existence of a level of host immunity able to restrict parasites to low, microscopically undetectable densities. Alternatively, these low-density infections may represent recently acquired infections which would reach microscopical levels if infection is not treated. Carriage of sub-microscopic infections has been shown to be common also in non-pregnant adults [30,31]. However, the occurrence of microscopically undetected infections are likely to be more clinically relevant during pregnancy, since malaria parasitaemia of any density may have a harmful effect on the pregnant women and her developing foetus [16,32,33].

In this study, the majority of women presenting clinical complaints suggestive of malaria were not infected with *P. falciparum*, as detected by microscopy (94.7%) and even by RT-PCR (76.8%). Thus, in this context, presumptive malaria treatment would lead to unnecessary exposure to anti-malarial drugs in the majority of pregnant women presenting with "malaria-like" clinical complaints [6]. On the other hand, treatment based on microscopically confirmed malaria episodes only, might lead to under-treatment because of the significant proportion of infections bellow the detection threshold of microscopy. The ability of RT-PCR to detect this additional population of women at risk for malaria infection that were otherwise microscopically negative would contribute effectively to a better management of malaria in pregnancy. However, PCR-based methods require costly reagents and instrumentation that are not widely available in developing countries. Rapid tests that detect parasite-specific antigens [7] may overcome the problem of inadequate diagnosis and treatment. In addition, a more sensitive definition of clinical malaria in pregnancy should also be evaluated. Other symptoms and signs could be considered, since those frequently used to define a malaria episode are poor indicators of *P. falciparum *infection both in pregnant women [6] and non-pregnant adults [31].

In this study, anaemia was the only symptom associated with parasitaemia as detected by RT-PCR. This association was not found for microscopical parasitaemia in this subset of samples, maybe due to limitations of the sample size. Women with sub-microscopic infections were also at higher risk of anaemia compared with women with negative microscopy and RT-PCR test, suggesting a role of low-density infections in the aetiology of maternal anaemia. A similar association has been found in some studies [31,34], but not in others [8,10,12,35], may be due to differences in the sensitivity of the molecular technique used. The results of this study suggest that low-density asymptomatic infections may significantly increase the risk of becoming anaemic. Alternatively, anaemia may be a consequence of recent high density infections controlled at the time of examination.

PCR-confirmed recrudescences were found in 21% of the women experiencing two successive episodes during the same pregnancy. The rate of recrudescences found in this study raises important questions for drug efficacy in pregnancy. First, it suggests that the efficacy of anti-malarial drugs during pregnancy may be reduced. This can be due to inadequate drug levels, limited clearing capacity of placental parasites, or to immunosuppresion and physiological changes during pregnancy which are likely to alter the pharmacokinetics of most anti-malarials [18-21,36]. Secondly, these results show that recrudescences of *P. falciparum *parasites during pregnancy can occur as late as 187 days. In other studies, reappearance of parasites has been shown to occur 35, 49, 85 and 119 days after treatment [37,38]. In Thailand, recrudescence was detected up to 62 days post-treatment in non pregnant individuals and up to 121 days in pregnant women [27]. Thus, the 28 days standard in vivo test [39] may not be adequate to assess the efficacy of anti-malarial drugs in pregnant women, since it would not detect recrudescences that occur much later. Various alternative assessment times have been used to define treatment failures in anti-malarial drug efficacy studies, ranging from 42 days [40-42] to 63 days [37,38] after treatment. This study suggests that even longer follow-up may be needed during pregnancy to capture all treatment failures. It is essential that these studies include molecular genotyping to distinguish recrudescences from reinfections.

## Conclusion

These results point out two important issues for malaria control during pregnancy; on the one hand, the need to apply more accurate and sensitive measures to detect malaria infections in pregnancy. On the other hand, it might be necessary to extend the follow-up period for in vivo tests to correctly identify anti-malarial drug failures during pregnancy. Appropriate, standardized genotyping methods should be applied to unequivocally identify recrudescent parasites. This is essential to optimize the drug regimens needed to eliminate the plasmodial biomass in pregnant women, and to correctly measure the efficacy of the existing and new anti-malarials to be used in pregnancy.

## Competing interests

The authors declare that they have no competing interests.

## Authors' contributions

AM and ES carried out the molecular genotyping study, the analysis and interpretation of data and prepared the manuscript. LP and PC contributed to the molecular genotyping study and the interpretation of data. SS and JA carried the statistical analysis and helped to draft the manuscript. AB and IM carried out the sample collection and contributed to the analysis and interpretation of data. BS contributed to the interpretation of the data and drafting of the paper. CM and PA conceived and coordinated the study, participated in the analysis and interpretation of the data, and contributed to the preparation of the manuscript. All authors read and approved the final manuscript.
